# Microsurgical Treatment Strategy of Vertebral Artery Fusiform Aneurysm—From the Standpoint of Hemodynamic Integrity and Perforator Preservation

**DOI:** 10.3389/fneur.2021.728176

**Published:** 2021-09-20

**Authors:** Sho Tsunoda, Tomohiro Inoue

**Affiliations:** Department of Neurosurgery, NTT Medical Center Tokyo, Tokyo, Japan

**Keywords:** fusiform aneurysm, vertebral artery, microsurgical treatment, hemodynamic integrity, perforator preservation, flow-augmentation bypass

## Abstract

During treatment of vertebral artery (VA) fusiform aneurysms, it is critical to preserve peripheral perforators and anterograde blood flow of the VA and to reduce hemodynamic load to the contralateral VA. Even in the era of endovascular treatment, there are still many benefits to using microsurgical treatments with appropriate clip application and preservation of the perforators around the aneurysm, in conjunction with various bypass techniques. The ideal microsurgical technique involves reconstructive clipping that obliterates the aneurysm but preserves anterograde blood flow of the VA, followed by isolation of the aneurysm and VA reconstruction. If these two methods are unavailable, proximal clipping of the aneurysm combined with flow-augmentation bypass to the distal branch can be considered as an alternative surgical management. We discuss the microsurgical treatment of unruptured VA fusiform aneurysms in our surgical cases on the basis of a review of the current literature.

## Introduction

The term “vertebral artery fusiform aneurysm” (VAFA) is generally used for spindle-shaped aneurysms that arise from the main trunk of the vertebral artery (VA). However, the clinical and pathological definition is ambiguous and, confusingly, the term is currently used for various types of aneurysms with different clinical and pathological features, including wide-neck saccular, dolichoectatic, and giant serpentine aneurysms. Many of the VAFAs reflect the consequences of dissecting changes at different time phases (1–3). Once rupture-induced subarachnoid hemorrhage (SAH) occurs, there is a high risk of devastating re-rupture in the acute phase of dissection. Thus, immediate treatment of SAH is required to preventing re-rupture (4–6). However, in patients with no rupture within the acute phase, the medium- to long-term natural history is unknown and there is no consensus regarding the treatment strategy.

Although endovascular treatment is now common for treatment of unruptured VAFAs, there are still many issues including risk of brainstem infarction (7–11). Furthermore, endovascular treatment cannot be used to treat revascularization in cases with branching arteries that are incorporated into the dissecting segment, such as the posterior inferior cerebellar artery (PICA) type. Thus, we emphasize the preservation of the VA branches and hemodynamic integrity for treatment of unruptured VAFAs, and ensure that we perform radical direct surgery with various bypass options. Herein, we report four cases of unruptured VAFA treated by our custom direct surgical approaches and discuss the treatment of unruptured VAFAs by direct surgery.

## Materials and Methods

### Patients' Characteristics

Between December 2017 and March 2021, we performed direct surgery on four cases (three men, one woman) with unruptured VAFAs (Table 1). The median age of the patients was 66.3 years (range, 55–77 years), the affected side was right in two cases, and the median aneurysmal size was 24 mm (range, 15–32 mm).

**Table 1:** Four cases of unruptured vertebral artery fusiform aneurysm treated by our custom direct surgical approaches.

**Case no**.	**Age**	**Sex**	**Affected side**	**Aneurysmal size (mm)**	**Classification of the aneurysm**	**PICA involvement**	**Treatment**	**Complications**
1	77	F	Right	32	Asymmetrically dilated fusiform	(-)	Reconstructive clipping	(-)
2	74	M	Left	15	Classic dissecting	(-)	Trapping, V3-RAG-V4 bypass	(-)
3	59	M	Right	19	Large thrombosed	(-)	Proximal ligation, V3-RAG-PICA flow-augmentation bypass	Small lateral medullary infarction (-)
4	55	M	Left	30	Giant thrombosed	(+)	Trapping, V3-RAG-V4 bypass, OA-PICA bypass	(-)

All patients received microsurgical treatment *via* a suboccipital far lateral approach with auditory brainstem response monitoring throughout the operation. All surgical procedures were performed by two specialized neurovascular surgeons (T.I. or S.T.). One asymmetrically dilated fusiform aneurysm (case 1) was treated by reconstructive clipping alone, two cases (cases 2 and 4) were treated by aneurysmal trapping with VA reconstruction using a V3 (extracranial VA)-radial artery graft (RAG)-V4 (intracranial VA) anastomosis, and one case (case 3) was treated by proximal ligation in conjunction with flow-augmentation bypass using V3-RAG-PICA anastomosis. Details of three representative cases are described below.

Written informed consent was obtained from the individuals for the publication of any potentially identifiable images or data included in this article.

### Case 1

**Figure 1:**
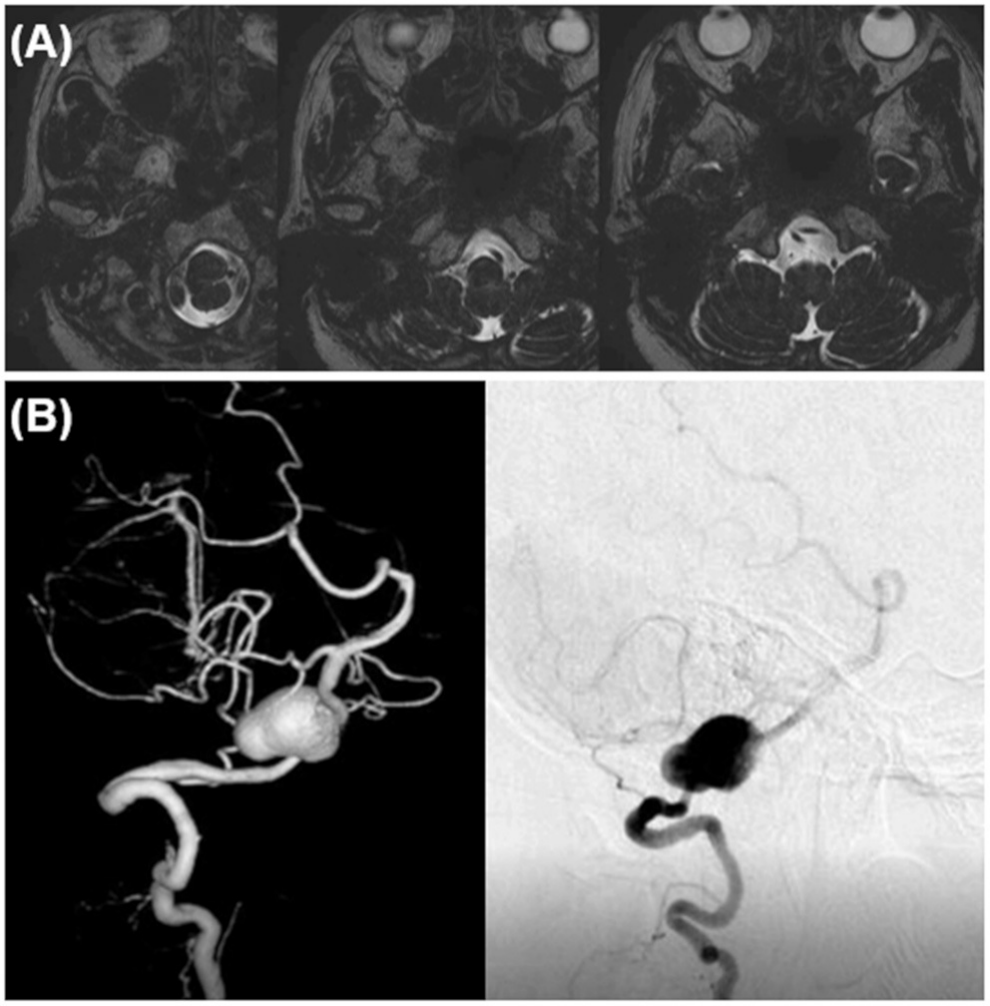
Preoperative image examinations of case 1. **(A)** Magnetic resonance imaging. **(B)** Digital subtraction angiography.

Case 1 was a 77-year-old woman. A right VA asymmetrically dilated fusiform aneurysm (14 × 32 mm) was incidentally found on brain magnetic resonance imaging (MRI) at checkup during treatment for otorhinolaryngologic disease. Preoperative MRI revealed that the proximal and distal necks were located at the height of the cerebellomedullary fissure and compressed the medulla oblongata from the anterior side (Figure 1A). On preoperative digital subtraction angiography (DSA), PICA originated from the VA just distal to the penetration part of the posterior dura, while no apparent branches from the aneurysm were identified (Figure 1B). The right VA was the non-dominant side, with atherosclerotic changes obvious in the affected VA and in all intracranial arteries. On the basis of the aneurysmal size and the patient's request, we decided to treat the lesion.

**Figure 2:**
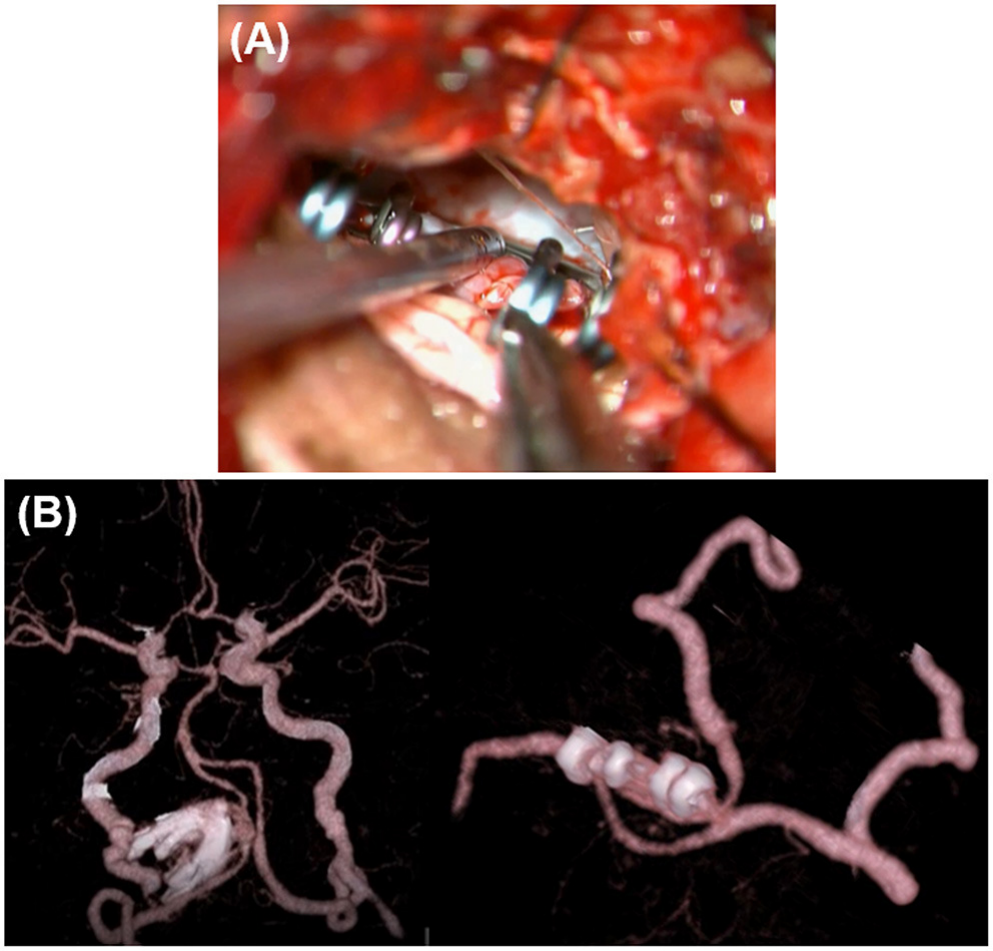
Intraoperative findings and postoperative computed tomography angiography (CTA) of case 1. **(A)** Intraoperative findings. **(B)** Postoperative CTA.

The dilated part mainly protruded outward and the entire aneurysm was localized around the cerebellomedullary fissure beneath the lower cranial nerves (LCNs). Thus, reconstructive clipping was considered anatomically and technically possible. During surgery, after confirming both the proximal and distal aneurysmal necks, we performed aneurysmal trapping with direct visualization and preservation of the peripheral perforators, and then incised the lateral wall of the aneurysm, decompressed the aneurysmal body, and obliterated the aneurysm by confrontation clipping with four clips (Figure 2A). The actual surgical procedures of case 1 is shown in Supplementary Video 1.

### Case 2

**Figure 3:**
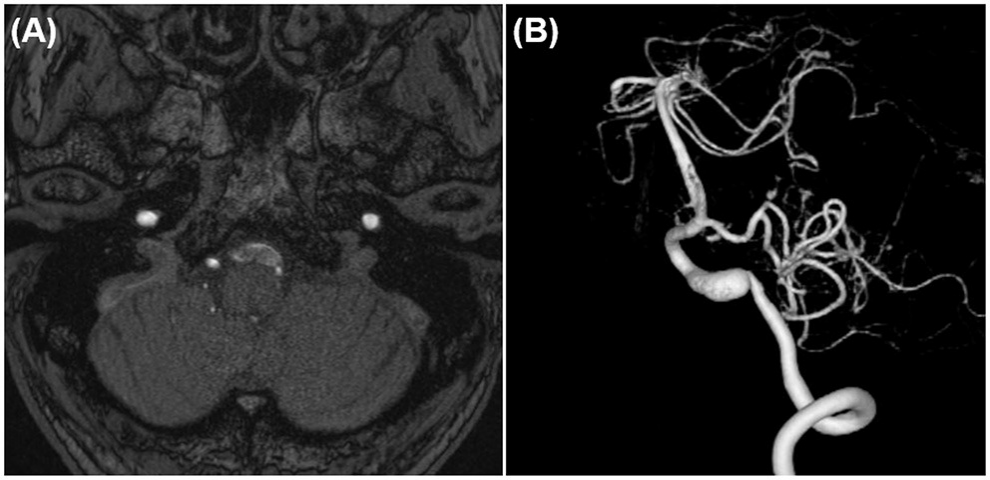
Preoperative image examinations of case 2. **(A)** Magnetic resonance imaging. **(B)** Digital subtraction angiography.

A 74-year-old man presented with sudden headache. A left VA dissecting aneurysm (15 × 6 mm) coexisting with a right internal carotid artery-posterior communicating artery aneurysm was found by MRI at a neighborhood clinic. He had a medical history of hypertension and heavy smoking (30 cigarettes per day for 40 years). On preoperative MRI, the dilated segment started at the height of the jugular foramen, ran transversely at the height of the 7–8th nerves toward the contralateral side, and ended at the point exceeding the midline (Figure 3A). The distal end was >20 mm proximal to the VA union. On preoperative DSA, the common trunk of the anterior inferior cerebellar artery (AICA)-PICA originated from the basilar artery, while the affected V4 had no angiographically identifiable branches (Figure 3B). The contralateral VA was as developed as the affected side. In addition to the irregular-shaped VA aneurysm in the subacute phase, there were other unruptured aneurysms. The patient also requested treatment to allow early return to his work (taxi driver). Thus, we decided to treat the left VA aneurysm at 2 months after onset.

**Figure 4:**
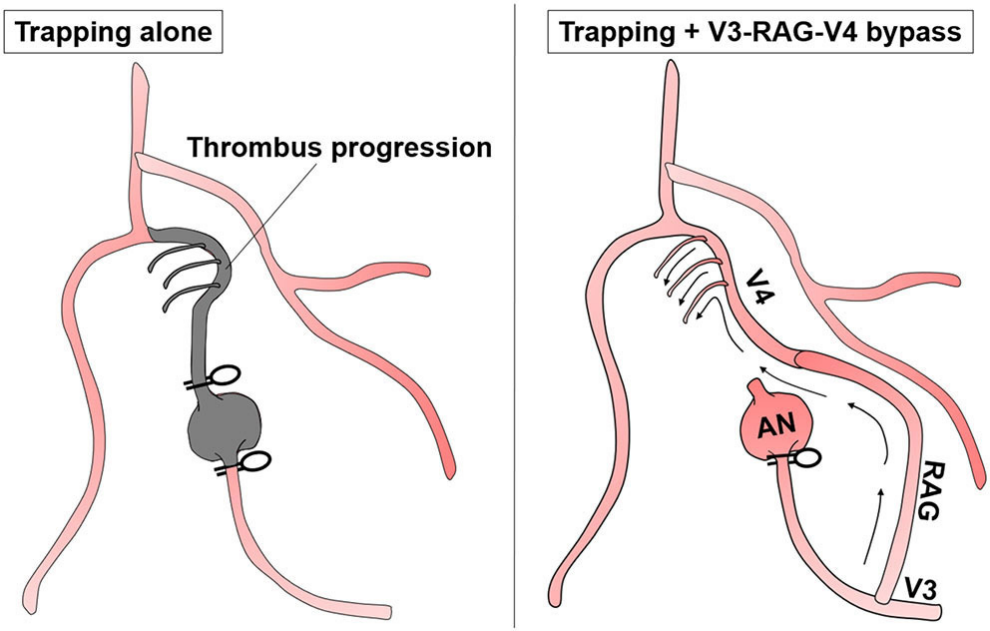
Schematic of the treatment strategy for case 2.

**Figure 5:**
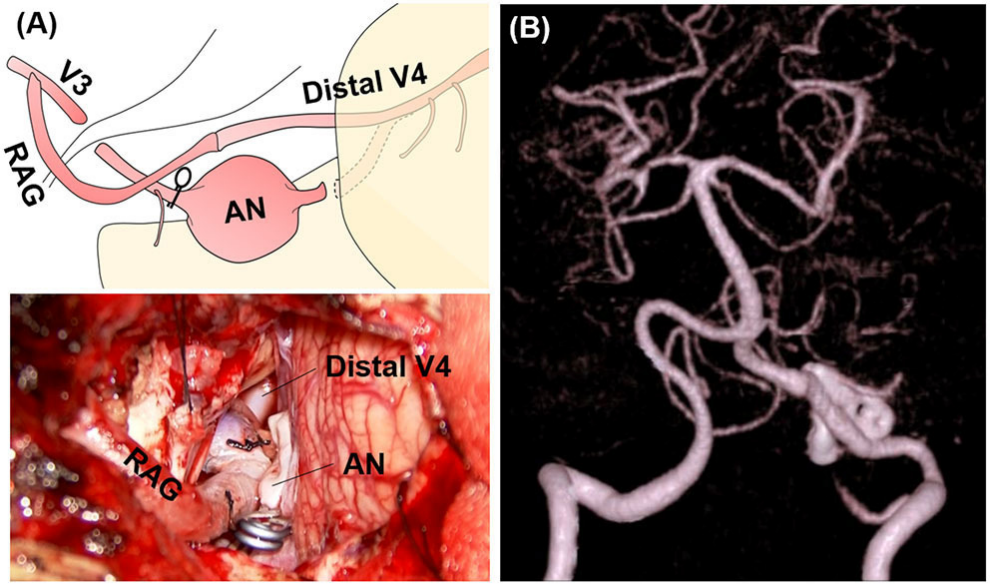
Intraoperative findings and postoperative computed tomography angiography (CTA) of case 2. **(A)** Intraoperative microscopic image and its schematic. **(B)** Postoperative CTA.

It was impossible to maintain the left VA by reconstructive clipping because of the aneurysm shape. However, it was possible to secure the distal neck and trap the aneurysm because it was located caudal to the 7–8th nerves. PICA revascularization was not necessary because the common trunk of the AICA-PICA was well-developed. Note that there was >20 mm space between the distal neck to the VA union. Furthermore, there was a concern that many brainstem perforators would be occluded because of thromboembolism after blinding of this segment by trapping. Thus, to preserve the anterograde blood flow of the VA, it was reconstructed using RAG to anastomose the V4 segment just distal to the aneurysm and the extracranial V3 segment (V3-RAG-V4 bypass) (Figures 4, 5A). The actual surgical procedures of case 2 is shown in Supplementary Video 2.

### Case 3

A 59-year-old man felt sudden headache and dizziness twice in a year. He visited a neighborhood clinic and a right VAFA (10 × 6 mm) was detected. Conservative therapy was selected. However, a third attack occurred with dysarthria and gait disturbance and he visited the emergency department of our hospital near his workplace. He had a medical history of hypertension and hyperlipidemia, and a heavy smoking history (30 cigarettes per day for 30 years).

**Figure 6:**
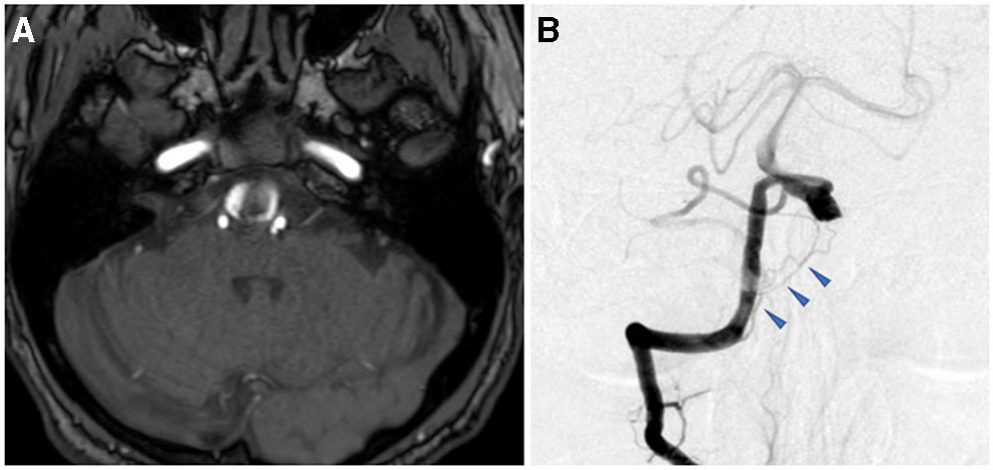
Preoperative image examinations of case 3. **(A)** Magnetic resonance imaging. **(B)** Digital subtraction angiography.

MRI after admission revealed expansion of the right VAFA (19 × 16 mm) that compressed the lower pons from the anterior side, while the intraluminal thrombus was increased compared with preadmission MRI (Figure 6A). Preoperative DSA showed that the dilated segment of the right VA started 12-mm proximal, and ended just proximal, to the VA union. The right side had a common trunk of the AICA-PICA, while the PICA was absent. However, there was a relatively thick branch (0.8 mm in diameter) feeding the lower vermis, lower pons, and medulla oblongata that arose from the V4 segment just distal to the penetrating part of the posterior dura (Figure 6B, blue arrowhead). In addition, no angiographically identifiable branch was found. The contralateral VA was well-developed.

**Figure 7:**
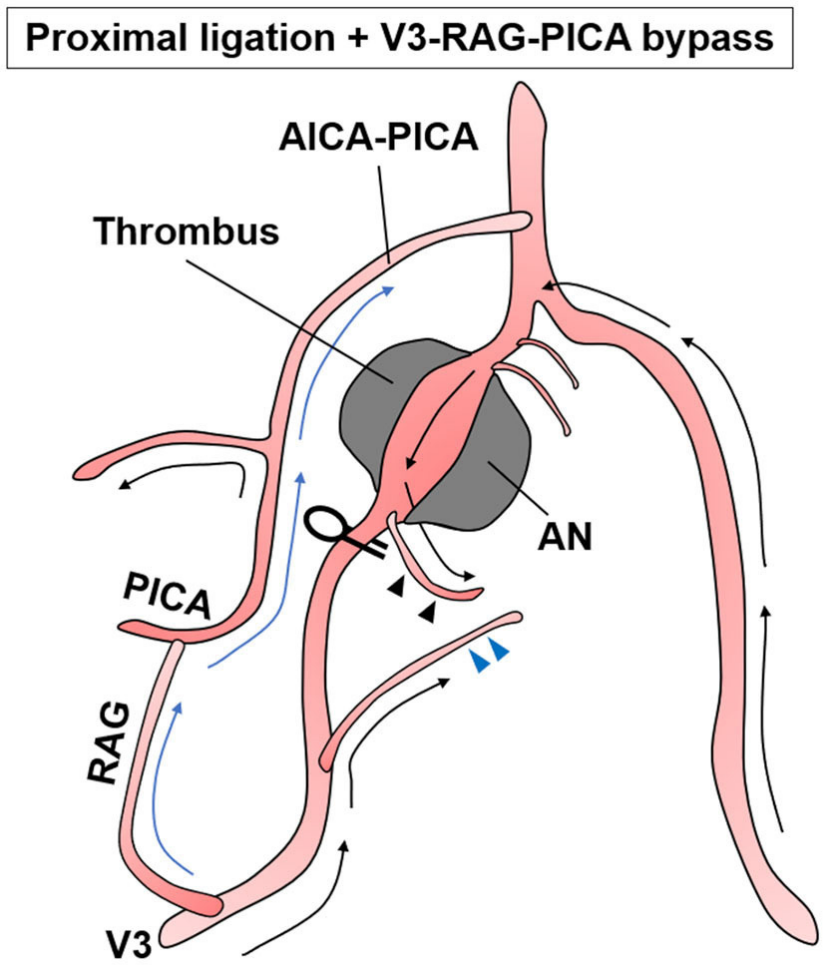
Schematic of the treatment strategy for case 3.

No other cause was found to explain his symptoms. Thus, brainstem compression caused by expansion of the thrombotic part of the aneurysm was considered the main cause. For treatment, the distal end of the aneurysm was positioned high and contralaterally displaced, making it difficult to perform reconstructive clipping as for case 1 or VA reconstruction as for case 2. Thus, we selected proximal ligation. The strategy was to reduce blood flow into the dilated segment of the aneurysm while maintaining the VA perforators by allowing the retrograde blood flow *via* the VA union to pass through the aneurysm and outflow to the VA perforator (Figure 7). In this situation, we had no choice but to make the distal segment of the ligation clip the so-called “perforator end,” which is hemodynamically prone to obstruction. Nevertheless, the potential for perforator obstruction was considered to be relatively low compared with aneurysmal trapping.

**Figure 8:**
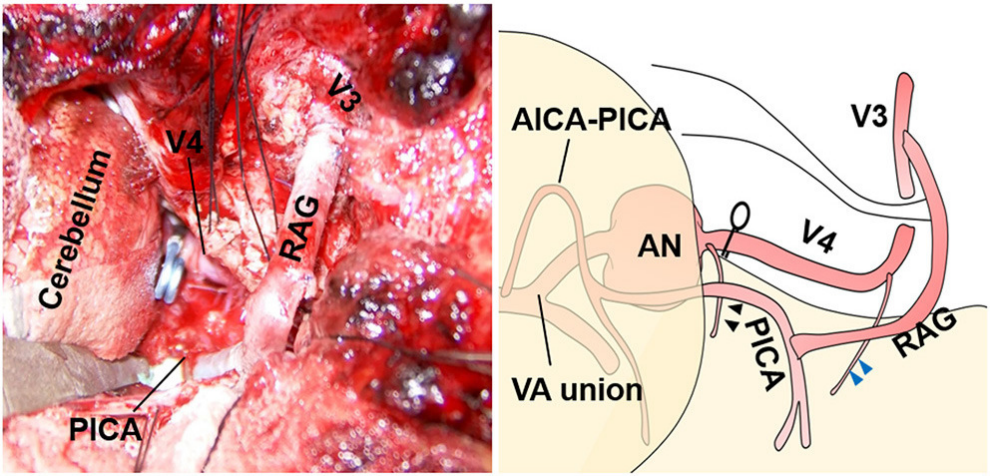
Intraoperative microscopic image and its schematic for case 3.

Intraoperatively, a perforator arose from the V4 segment just proximal to the aneurysm and there was no distance for clip application between the proximal neck to this perforator (Figures 7, 8, black arrowhead). Thus, the proximal portion of this perforator was ligated while ensuring visually that there was no other conspicuous VA perforator. The anterograde blood flow of the VA was allowed to flow out to the aforementioned relatively thick branch from the V4 (Figures 7, 8, blue arrowhead). Additionally, a flow-augmentation to the right PICA through the V3-RAG-PICA bypass secured the perfusion of the right AICA-PICA region to reduce the hemodynamic load on the contralateral VA. This was expected to be a detour to substitute for the VA trunk (Figure 7, blue arrow) in case the contralateral VA become occluded in the future. The actual surgical procedures of case 3 is shown in Supplementary Video 3.

## Results

**Figure 9:**
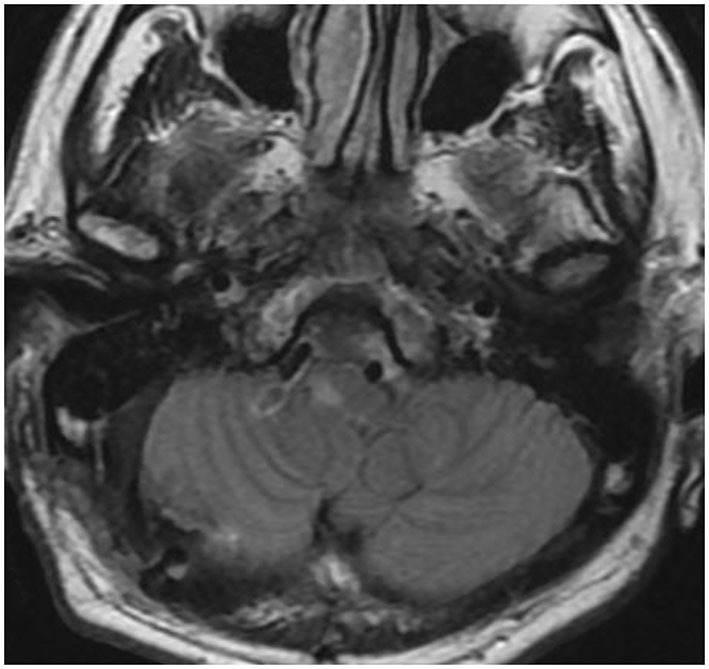
Postoperative fluid-attenuated inversion recovery imaging of case 3 revealed a small lateral medullary infarction.

**Figure 10:**
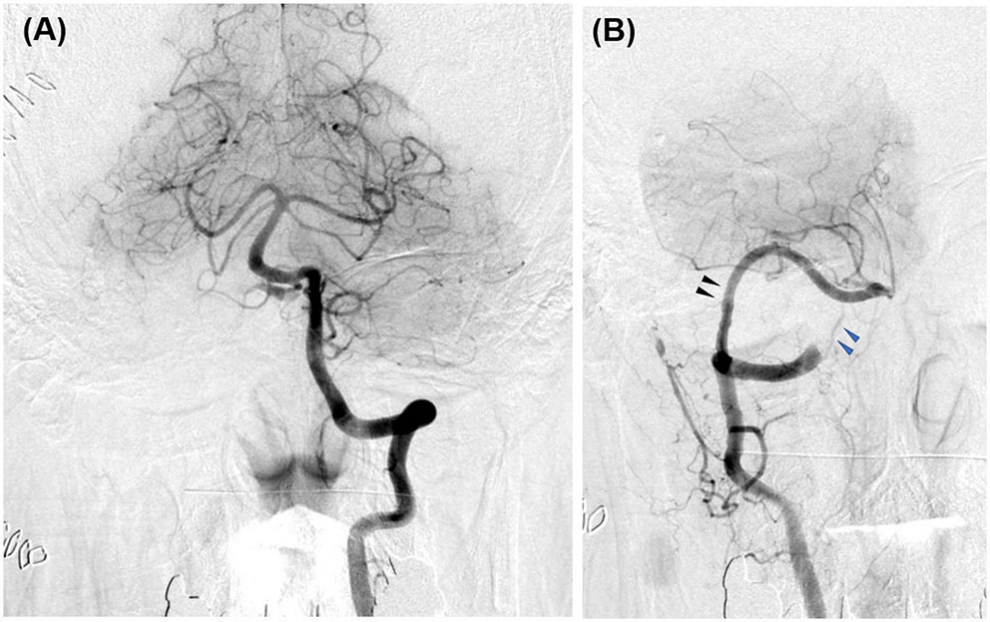
Postoperative digital subtraction angiography of case 3. **(A)** Left vertebral angiography (VAG). **(B)** Right VAG. The V3 (extracranial VA)-radial artery graft (RAG)-posterior inferior cerebellar artery (PICA) bypass broadly fed the anterior inferior cerebellar artery-PICA region (black arrowhead). The flow of the VA main trunk went into the relatively thick branch feeding the lower vermis and the medulla oblongata (blue arrowhead).

In case 1, 2, and 4, the aneurysms were completely obliterated and good reconstruction of the VA was confirmed (Figures 2B, 5B). There were no complications, such as postoperative infarction, cranial nerve palsy, or cerebrospinal fluid leakage, in these cases. However, a small right-sided lateral medullary infarction appeared in case 3 (Figure 9), with complications of dissociated sensory disturbance, dysphasia, and latero-pulsion. Nevertheless, all symptoms apart from the sensory disturbance improved after 1 month of rehabilitation. On postoperative DSA, retrograde blood flow *via* the VA union passed through the parent artery of the aneurysm and flowed out to the VA perforator, while the inflow into the dilated part of the aneurysm disappeared (Figure 10A). As planned, the anterograde blood flow of the VA trunk flowed out to the thick VA branch (Figure 10B, blue arrowhead). The PICA and AICA regions were fed *via* the V3-RAG-PICA bypass (Figure 10B, black arrowhead), confirming a reduction in the hemodynamic load on the contralateral VA.

## Discussion

### Treatment Timing of the Unruptured VAFA

Many VAFAs are considered to occur following dissecting changes such as extension and fragmentation of the internal elastic lamina due to hemodynamic or mechanical stress, subsequent blood inflow between the arterial walls, compensatory intimal thickening mainly composed of collagen fibers, and various arterial form changes due to their degeneration and collapse (12–15).

If this thickened intima is stable, aneurysm growth and morphological changes will stop, whereas when the thickened intima is unstable, it can rupture to generate a thrombus in the dissecting cavity. As the thrombus regresses, blood flows into the outside of the thrombus, resulting in extension of the adventitia and media and repeated aneurysmal growth (16, 17).

The long-term prognosis of such chronic type aneurysms is unknown. Giant aneurysms (>25 mm) were previously reported to be often spontaneously thrombosed and healed, whereas their overall rupture rate and late (5-year) mortality rate were reported as 60–80% and 80%, respectively (18). Hence, more recently, treatment is often considered when the aneurysm size exceeds 10 mm (19, 20). Moreover, if the dilated part of the aneurysm continues to grow, brainstem compression by the aneurysm and thromboembolic events caused by the intra-aneurysmal thrombosis may occur, which can lead to catastrophic rupture. Thus, treatment should be considered when these signs are observed (21, 22). We decided to treat cases 1 and 2 based on the aneurysm size and shape and cases 3 and 4 based on the increase in the aneurysm size over time and the brainstem compression symptoms.

### Treatment Selection

Although direct surgery is widely used for treatment of aneurysms (23), the last decade has seen increased use of endovascular approaches for first-line treatment.

Endovascular treatment using internal trapping is considered effective for non-branching types of aneurysms (24). However, the potential for post-procedural cranial nerve palsy, perforator infarction, and spinal cord infarction is also high (8, 25). Sacrificing one of the two VAs that feed vital brain structures such as the brainstem and cerebellum should be avoided if possible. Thus, reconstructive treatment of the unruptured VA aneurysm including stent-assisted coiling or a flow diverting stent is increasingly used as a substitute for internal trapping (26–31). Nevertheless, there are contrasting findings on the use of stents for reconstructive treatment, with reports of either no procedural complications or of higher complication rates in stent-assisted treatment compared with the simple technique (10, 11). As for flow diverting devices, the procedure-related complication rate for treatment of posterior circulation aneurysms was reported to be as high as 25%, while the complete occlusion rate was as low as 35–75% (32–34). Moreover, there are no cohesive reports on the patency of the branches such as AICA, PICA, and brainstem perforators over the longer term. Overall, there is no current consensus on the medium- to long-term outcomes of these reconstructive endovascular treatments.

By contrast, although there is a risk of cranial nerve palsy, direct surgery can provide an appropriate site for clip application while allowing comprehensive observation of the branching arteries in the vicinity of the aneurysm. This reduces the risk of postoperative infarction due to branch occlusion compared with endovascular treatment. Direct surgery can also be performed for various revascularizations. Thus, we prefer to use direct surgery. Our treatment principles for direct surgery are described below.

### Principles in Direct Surgery for VAFA

D'Ambrosio et al. reported that for approaching the VA-PICA region while minimizing the risk of LCN palsy, a trajectory from the caudo-dorsal side of the vulnerable LCNs should be used, and that the next most useful corridor is the space between the LCNs and 7–8th nerve complex (35–37). To use these two corridors from a more caudo-dorsal direction, we utilize the suboccipital far lateral approach. Prior to this procedure, we perform a layer-by-layer dissection of the suboccipital muscles, which allows us to systematically identify the occipital artery (OA), extracranial VA (V3 portion) in the suboccipital triangle, and the posterior condylar canal. These steps enable the OA-PICA bypass, proximal control by temporary clamping of the V3, and high-flow bypass using the V3 as a blood source (38–40). The actual procedural steps are shown in Supplementary Video 1.

The principle for this treatment is to achieve reconstructive clipping, as presented in case 1. However, an appropriate site for clip application, avoiding kinking of the parent artery due to clipping (no calcification or intraluminal thrombus), and firm margin of the aneurysmal wall that can be clipped are essential. Furthermore, decompression of the aneurysm (suction decompression and thrombectomy) and ingenious clipping (combination clipping or clipping on wrapping) are often required (41–43). It is often difficult to perform these procedures *via* the narrow space where the dense cranial nerve bundles are located. Thus, deconstructive measures are mostly used.

The principle of the deconstructive method involves isolation of the aneurysm by aneurysmal trapping. In particular, in cases with a giant thrombosed aneurysm, micro-bleeding from the vasa vasorum in the aneurysmal wall is considered a cause of aneurysm growth (44, 45). Thus, complete isolation of the aneurysm by trapping is desired for obstruction of the vasa vasorum. If trapping is difficult because the distal end cannot be secured anatomically, the aneurysm should be turned into a blind end while preserving the VA perforator and the anterior spinal artery to the extent possible. VA aneurysms can be divided into three basic types: post-PICA, pre-PICA, and PICA-involved. The post-PICA and pre-PICA types can be turned into a blind end using proximal clipping (2, 46). However, for the PICA-involved type or other post-PICA types in which the distance between the aneurysm and the PICA origin is small, revascularization of the PICA is required according to the situation (4, 47). Regarding revascularization of the PICA, OA-PICA (48, 49), PICA-PICA (50), VA-superficial temporal artery-PICA (51), VA-RAG-PICA (52), and PICA reimplantation (53) have been reported. However, most groups including our own prefer to use the OA-PICA bypass, except for cases where it is impossible to use the OA.

### Preservation of Brainstem Perforator and Revascularization of the Affected VA

When the deconstructive method is used, thrombus progression may occur in the VA proximal to the blind-ended aneurysm. This results in the so-called “VA stump syndrome,” whereby the VA branches including the brainstem perforator and the anterior spinal artery are involved and occluded (54, 55). However, it was reported that even if the VA is blinded, perforating branch infarction does not occur in the presence of an anterior spinal artery (0.7–0.8 mm in diameter) (56). Furthermore, a report on trapping using an external carotid artery-M2 bypass for blister-like aneurysms of the internal carotid artery showed that outflow into an adult-type posterior communicating artery or anterior choroidal artery should be avoided (57). In other words, the risk of obstruction of the branches proximal to a relatively robust outflow vessel is low (58). In proximal clipping or trapping of the VAFA, the VA perforators often arise from within 10 mm of the VA union (58). Thus, the surgical strategy should be designed to keep this portion from becoming a blind end.

In cases of the pre-PICA type, blinding of this portion can be avoided because the retrograde blood flow *via* the VA union flows out to the PICA, even if proximal clipping or aneurysmal trapping is performed. However, in cases of the PICA-involved, post-PICA, or non-branching types of aneurysms, proximal ligation or aneurysmal trapping can blind the VA distal to the aneurysm and occlude it by thromboembolism. In such cases, VA reconstruction should be performed to the extent possible to maintain the anterograde blood flow of the distal V4 of the affected side.

In case 2 (Figure 4), the space between the aneurysm and the VA union was >20 mm and we predicted that the branches including the brainstem perforators were concentrated at that site. Because there was no visible outflow vessel on preoperative DSA, the use of only trapping had the potential to cause VA stump syndrome and obstruction of all the VA perforators. For these reasons, we decided to preserve the anterograde blood flow of the VA distal to the trapped aneurysm by using V3-RAG-V4 bypass.

In case 3, the distal end of the VA aneurysm was positioned high, just before the union, while the section 10 mm before the union (which is considered to have many perforators) was incorporated into the aneurysm. Anatomically, reconstructive clipping and VA reconstructive bypass (as for case 2) were unavailable. Thus, proximal ligation was chosen. Proximal ligation for the non-branching type of aneurysm involves making the distal segment of the ligation clip the “perforator end,” which is hemodynamically prone to obstruction. However, unlike coil occlusion, in direct surgery the thrombogenic coil is not exposed in the intravascular lumen and the vessel wall at the blind end is rebuilt by the clip, which maintains endothelial continuity. Thus, despite the occurrence of a “perforator end,” the risk of perforator obstruction by thrombus progression caused by VA stump syndrome is less than that for endovascular coiling (25). In addition, a relatively thick perforator was found at the proximal end of the aneurysm. Therefore, VA stump syndrome was avoided by attenuating blood flow to the aneurysm using proximal ligation and by draining the retrograde blood flow *via* the VA union into this VA perforator (Figure 7). In case 3, development of the small lateral medullary infarction was likely related to obstruction of the VA perforator, which was involved in the blinded VA thrombosis. Nevertheless, the damage was minimized because the best clipping site was selected. As this strategy has potential for unexpected postoperative infarction, it should only be used in cases where a “perforator end” is unavoidable.

The majority of patients with VA aneurysms show coexistence of atherosclerotic vascular disease in other vessels (~70%) and pathological contralateral VA dissection (~40%). Thus, there is no guarantee of permanent patency of the contralateral VA (2, 59). Furthermore, increased hemodynamic load to the contralateral VA after occlusion of the affected VA may cause a higher risk of contralateral VA dissection (60). Thus, it is essential to preserve the affected VA and reduce hemodynamic load to the contralateral VA as much as possible. As such, the ideal method is reconstructive clipping as described in case 1, or V3-RAG-V4 bypass to preserve the VA anterograde blood flow when reconstructive clipping is not possible, as shown in case 2.

As we previously reported (39), the V3-RAG-V4 anastomosis procedure can be safely performed in shallow surgical fields if the distal V4 segment can be pulled down to the triangular space below the LCNs. In particular, in the case of giant VA aneurysms the distal V4 segment is often meandering and deflects outwardly to the vicinity of the internal auditory canal, while the distal V4 and branches of the VA are often stretched in an axial direction. Thus, the V4 stump can be easily transposed to the triangle corridor beneath LCNs without perforator injury (37, 39, 60, 61). If reconstructive clipping is impossible and a V3-RAG-V4 bypass is difficult, then VA occlusion alone is insufficient. Thus, it is important to preserve the brainstem perforators and maintain the hemodynamic integrity to the extent possible.

In case 3, the flow-augmentation bypass of the V3-RAG-PICA anastomosis was performed for two reasons. First, this bypass was expected to supply the perfusion of the right AICA-PICA area, resulting in reduced hemodynamic load on the contralateral VA. Second, this bypass was expected to be a detour to substitute for the VA trunk (Figure 7, blue arrow), in case the contralateral VA is accidentally occluded in the future. Although there are pros and cons to the safety of this bypass procedure, a stable RAG-PICA anastomosis is possible if a thick PICA can be secured in the cerebellomedullary fissure and the surgeon can perform an anastomosis technique in the deep corridor. If reconstructive clipping or VA reconstruction are unavailable, a flow-augmentation bypass to the distal branch can be considered as an alternative method.

## Conclusion

Preservation of the peripheral perforators and anterograde blood flow of the VA, and reducing hemodynamic load to the contralateral VA, are essential for VAFA treatment. Furthermore, radical surgical treatment to achieve perforator preservation and hemodynamic integrity, including thorough treatment strategies, appropriate clip application methods, and various bypass procedures, are critical to ensure the medium- to long-term good outcomes while reducing the potential for postoperative complications.

From the above, even in the era of endovascular treatment, there are still many benefits to using microsurgical techniques in the management of VAFA. However, the sample size of this study was small and the characteristics of VAFA is highly diverse, thus the result of this study should not be generalized.

## Data Availability Statement

The original contributions generated for the study are included in the article/Supplementary Material, further inquiries can be directed to the corresponding author/s.

## Ethics Statement

The studies involving human participants were reviewed and approved by the ethics committee of NTT Medical Center Tokyo. The patients/participants provided their written informed consent to participate in this study. Written informed consent was obtained from the individuals for the publication of any potentially identifiable images or data included in this article.

## Author Contributions

ST and TI: conceptualization, methodology, and data investigation. ST: writing original draft preparation. TI: review and editing. All authors contributed to the article and approved the submitted version.

## Conflict of Interest

The authors declare that the research was conducted in the absence of any commercial or financial relationships that could be construed as a potential conflict of interest.

## Publisher's Note

All claims expressed in this article are solely those of the authors and do not necessarily represent those of their affiliated organizations, or those of the publisher, the editors and the reviewers. Any product that may be evaluated in this article, or claim that may be made by its manufacturer, is not guaranteed or endorsed by the publisher.

## Supplementary Material

Supplementary Video 1 This video clip demonstrates the actual surgical procedures used in our case 1. Case 1 (asymmetrically dilated fusiform aneurysm) was treated by reconstructive clipping alone. In this video, the layer-by-layer dissection of the suboccipital muscle and far-lateral drilling is shown.

Supplementary Video 2 This video clip demonstrates the actual surgical procedures used in our case 2. Case 2 was treated by aneurysmal trapping with VA reconstruction using V3 (extracranial VA)-radial artery graft (RAG)-V4 (intracranial VA) anastomosis.

Supplementary Video 3 This video clip demonstrates the actual surgical procedures used in our case 3. Case 3 was treated by proximal ligation in conjunction with flow-augmentation bypass using V3-RAG-posterior inferior cerebellar artery anastomosis.
